# Annexin-enriched osteoblast-derived vesicles act as an extracellular site of mineral nucleation within developing stem cell cultures

**DOI:** 10.1038/s41598-017-13027-6

**Published:** 2017-10-03

**Authors:** O. G. Davies, S. C. Cox, R. L. Williams, D. Tsaroucha, R. M. Dorrepaal, M. P. Lewis, L. M. Grover

**Affiliations:** 1 School of Sport, Exercise and Health Sciences, Loughborough University, Epinal Way, Loughborough, LE11 3TU UK; 2 School of Chemical Engineering, University of Birmingham, Edgbaston, Birmingham, B15 2TT UK; 3 UCD School of Biosystems and Food Engineering, University College Dublin, Belfield, Dublin, 4 Ireland

## Abstract

The application of extracellular vesicles (EVs) as natural delivery vehicles capable of enhancing tissue regeneration could represent an exciting new phase in medicine. We sought to define the capacity of EVs derived from mineralising osteoblasts (MO-EVs) to induce mineralisation in mesenchymal stem cell (MSC) cultures and delineate the underlying biochemical mechanisms involved. Strikingly, we show that the addition of MO-EVs to MSC cultures significantly (P < 0.05) enhanced the expression of alkaline phosphatase, as well as the rate and volume of mineralisation beyond the current gold-standard, BMP-2. Intriguingly, these effects were only observed in the presence of an exogenous phosphate source. EVs derived from non-mineralising osteoblasts (NMO-EVs) were not found to enhance mineralisation beyond the control. Comparative label-free LC-MS/MS profiling of EVs indicated that enhanced mineralisation could be attributed to the delivery of bridging collagens, primarily associated with osteoblast communication, and other non-collagenous proteins to the developing extracellular matrix. In particular, EV-associated annexin calcium channelling proteins, which form a nucleational core with the phospholipid-rich membrane and support the formation of a pre-apatitic mineral phase, which was identified using infrared spectroscopy. These findings support the role of EVs as early sites of mineral nucleation and demonstrate their value for promoting hard tissue regeneration.

## Introduction

Bone fractures present a growing worldwide medical and socioeconomic burden, with 8.9 million reported annually solely as a result of osteoporosis^1^. Despite the natural regenerative capacity of bone, there are instances where healing is impaired and clinical intervention becomes essential. Examples include when the quantity of bone required is simply beyond the body’s natural regenerative capacity, such as in the case of critical sized bone defects resulting from trauma or invasive surgeries (e.g. osteosarcoma excision), delayed or non-unions, or when the natural regenerative capacity is impaired due to osteoporosis or avascular necrosis^2^. Standard clinical approaches currently used to stimulate or augment bone regeneration include distraction osteogenesis and bone transport, autologous or allogeneic bone grafts, or the application of bone graft substitutes (BGS) sometimes combined with hyper-concentrated growth factors, such as bone morphogenetic proteins (BMPs) – for example INFUSE^®^ grafts^3^. Although these methods are used in clinical practice with positive results, each suffers from significant limitations and even promising osteoinductive approaches utilising the growth factor BMP-2 have been subject to controversy and serious negative outcomes^4^. This means that although current interventions offer a valuable method to facilitate bone repair, none of these approaches can be considered optimal and potent methods of inducing osteogenesis that are able to rapidly generate bone without associated patient morbidity are required^5^.

Modern tissue engineering approaches for hard tissue formation have been the subject of substantial research over the past two decades, with recent advances in therapies that combine osteoconductive materials with cells providing novel ways of promoting osteogenesis^6^. Cell-based approaches are appealing since they attempt to recapitulate and exploit the body’s natural regenerative capacity and to date there have been a number of significant advances made in orthopaedics in this area^7^. However, it has become increasingly clear that the considerable benefits offered by cell-based approaches will be difficult to translate into clinical practice since progression is frequently hindered by significant and sometimes insurmountable hurdles associated with ethics, government regulation, and high associated costs^8^. With this in mind, there is considerable merit in developing new biological methods of bone regeneration that retain the considerable benefits of a cell-based approach.

Many of the beneficial effects once attributed to cells are now thought to be, at least in part, a consequence of paracrine factors packaged within extracellular vesicles (EVs)^9^. Over the past decade, the importance of EVs in cell-cell communication and tissue regeneration has become increasingly recognised. With this in mind, it has been recently suggested that the use of EVs for regenerative medicine could be the next logical progression in the field^10,11^. Nowhere is the critical developmental role of EVs more obvious than in the skeletal system, where EVs have historically been associated with sites of early mineral formation^12,13^. EVs act as means of mediating communication between osteoblasts and osteoclasts to maintain bone homeostasis. As such, vesicular trafficking is important during bone modelling and remodelling, with osteoblasts shown to use vesicles for the transport of RANKL to osteoclast precursors to stimulate osteoclast formation^14^. Similarly, osteoclast-derived EVs have been implicated as inhibitors of osteoblast activity through the transfer of miRNA^15^ as well as paracrine regulators of osteoclastogenesis, possibly through competitive inhibition of RANKL^16^. At present, the application of EVs as a therapeutic vehicle for the delivery of regeneration-enhancing biological cargos is only just becoming apparent^17^. The proposition of applying EVs for regenerative medicine presents considerable benefits over traditional cell-based approaches, with conversion upon implantation. In addition, there are thought to be fewer immunological risks associated with the delivery of EVs, due to the fact that induced pluripotent stem cell-derived exosomes have previously been shown to lack MHC class I and II proteins^18,19^. Furthermore, much like stem cells or osteoinductive growth factors, EVs have the capacity to be incorporated into materials-based delivery systems to enhance tissue formation^20^. Given the potential benefits of EVs, it is surprising that, to date, only a limited number of studies have sought to utilise these valuable factors for regenerative medicine applications^21,22^. We propose that there is considerable utility in the application of osteoblast-derived EVs as a source of osteoinductive components that can promote osteogenesis for hard tissue regeneration.

In this study we analysed the potential for osteoblast-derived EVs to act as acellular biological vehicles for the delivery of pro-osteogenic cargos able to induce mineralised tissue formation in human bone marrow stem cell (hBMSC) cultures. Proteomic analysis of EVs derived from mineralising (MO-EVs) and non-mineralising osteoblasts (NMO-EVs) was performed to define how culture status influenced their resulting biological contents. By administering EVs to hBMSC cultures we determined how these biological differences influenced their pro-osteogenic effects and compared them with the current gold-standard, BMP-2. Finally, by applying a combination of biological and advanced physical techniques we were able to probe the fundamental mechanisms underlying EV-mediated mineralisation within the developing extracellular matrix to identify these vesicles as sites of mineral nucleation and development.

## Results

### Extracellular vesicles can be isolated from the culture medium of mineralising osteoblasts

**Figure 1:**
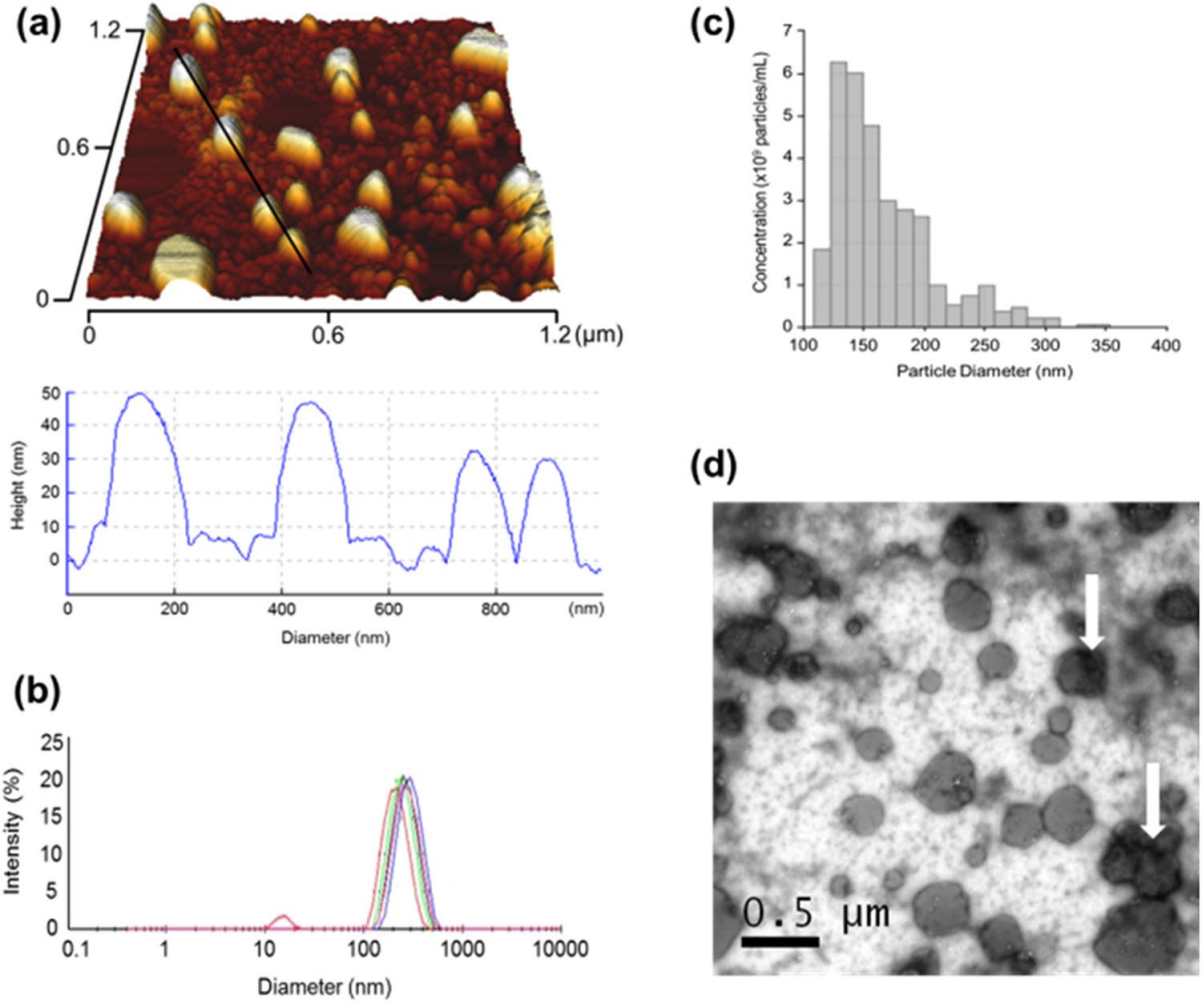
Profiling and characterisation of sEVs derived from mineralising osteoblasts (MO-EVs). (**a**) Topographic profiling of sEVs using AFM under tapping mode revealed a heterogeneous population of spherical particles with sizes within the range previously obtained for osteoblast EVs (<300 nm). (**b**) Tunable resistive pulse sensing measurements revealed a mean particle size of 167 nm, with a maximum particle size of 340 nm. (**c**) DLS measurement also confirmed a largely mono-disperse population with a mean particle size of 157 nm. (**d**) Transmission electron micrograph identifying a heterogenous population of sEVs with white arrows identifying the presence of what appear to be membrane-associated condensations associated with mineral accumulation.

EVs were isolated using differential centrifugation from the spent culture medium of mineralising osteoblasts (MO) at two day intervals over a total period of 14 days, as previously described^23^. The presence of EVs was first confirmed by immobilising vesicles on negatively charged mica surfaces for atomic force microscopy (AFM) analysis. Intact nano-particles (Fig. 1a) that could be immobilised electrostatically and presented a size range of between 79–171 nm were identified using AFM. Particles did not all appear spherical, likely due to previously documented surface interactions occurring between the EVs and the mica surface^24^. Direct Light Scattering (DLS, Malvern Instruments), tuneable resistive pulse sensing (IZON qNano) and nanoparticle tracking analysis (Nanosight, Malvern Instruments, Supplementary information S1) confirmed the presence of EVs with a mean diameter below 200 nm, correlating with diameters previously documented for exosomes and excluding the presence of apoptotic bodies and any larger components of the micro-vesicle fraction^25^. Average measurements of 157 nm (DLS) and 167 nm (Izon qNano) were obtained (Fig. 1b and c), which highlighted previously recognised variation between these methods related to their methodology as well as heterogeneity within the final EV fraction. The fraction was largely mono-disperse, with a bell shaped curve recording particle sizes between 30–340 nm. The presence of particles above the documented size of exosomes was noted but observed sizes were in line with bone-derived vesicles previously published in the literature (≤300 nm)^26^. In order to not further growing confusion regarding EV definitions within the literature, this likely heterogeneous population of exosomes and small micro-vesicles are referred to non-specifically as small extracellular vesicles (sEVs), based on a previous designation^27^. Transmission electron microscopy further demonstrated the presence of sEVs with diameters above and below the mean particle size (Fig. 1d). sEVs also exhibited heterogeneity in both EV diameter and the presence of electron dense regions, which were observed primarily in the region of the cell membrane (Fig. 1e, white arrows), suggesting localised mineral aggregation.

### sEVs derived from mineralising osteoblasts enhanced the differentiation of human bone marrow mesenchymal stem cells when compared with the current gold standard

We sought to determine whether electron-dense sEVs derived from mineralising osteoblasts, henceforth referred to as mineralising osteoblast-derived extracellular vesicles (MO-EVs), were able to promote mineralisation when added to hBMSC cultures, and how these compared against sEVs derived from non-mineralising osteoblasts (NMO-EVs), a positive control (mineralisation medium only) and the current gold-standard, BMP-2. For the generation of sEVs, cell seeding density, culture time and dosage were constant between all treatments.

**Figure 2:**
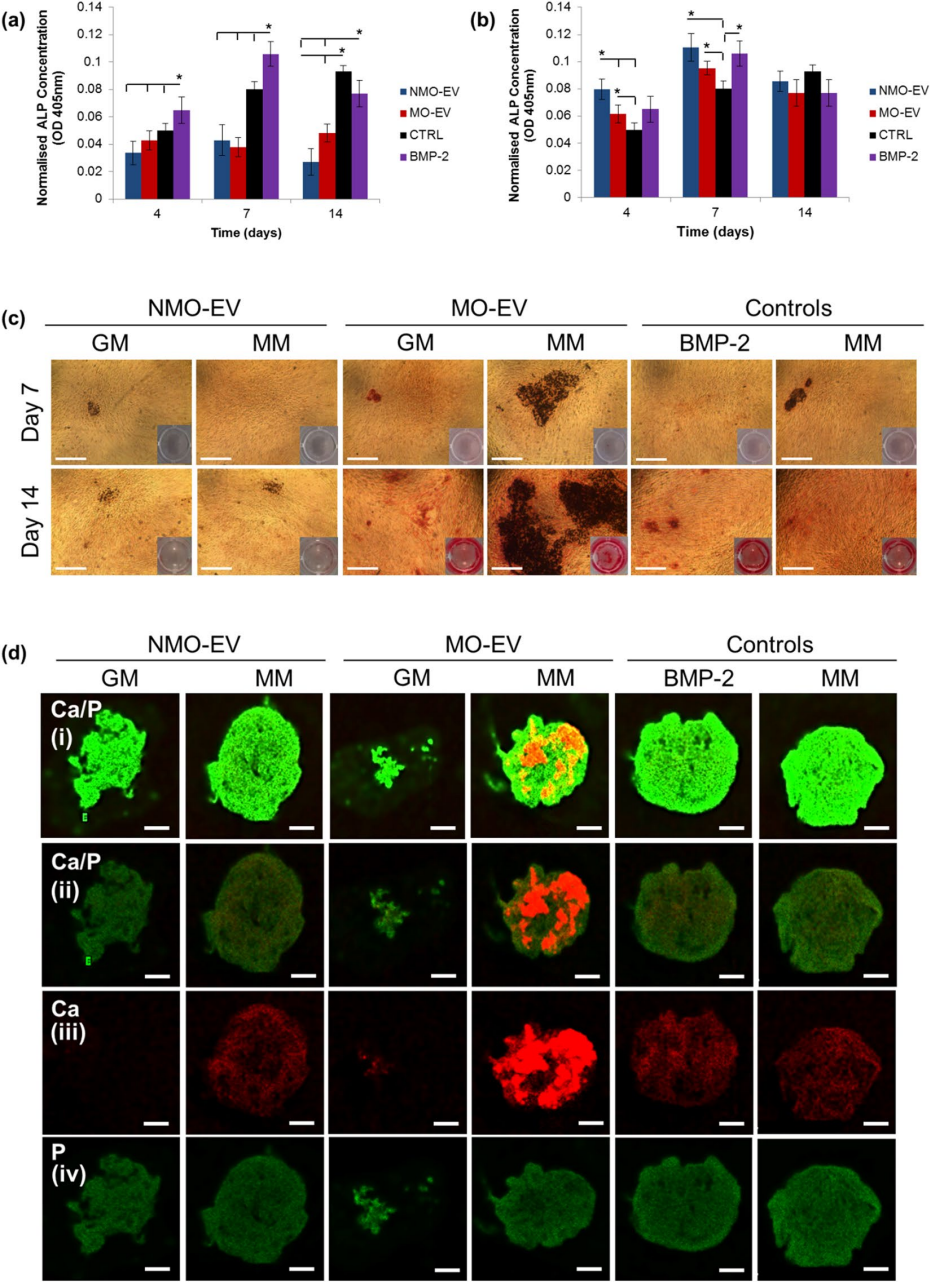
Examining the osteoinductive effects of MO-EVs on whole hBMSC cultures when compared with the current gold-standard, BMP-2. For all experiments the osteoinductive effects of MO-EVs were evaluated in growth medium and mineralisation medium (MM). MO-EVs were compared with NMO-EVs and BMP-2. ALP levels were measured at days 4, 7, and 14 in the presence of (**a**) growth medium (GM) and (**b**) mineralisation medium (MM). ALP levels were significantly elevated in MO-EV and NMO-EV treated cultures at days 4 and 7 only in the presence of MM. (**c**) Alizarin red calcium staining of hBMSC cultures at days 7 and 14 of culture with corresponding whole plate images displayed in the bottom right hand corner. Comparatively large areas of calcium deposition were observed in the hBMSC cultures treated with MO-EV/MM when compared with all other treatments. (**d**) X-ray fluorescence (XRF) elemental mapping confirmed the presence of comparatively large areas of calcium (red) deposition in MO-EV/MM treated cultures. XRF images are provided in (i) relative mode to distinguish areas of Ca (red), P (green) and co-localisation (yellow), and (ii) absolute mode to best represent how much Ca and P contribute to the total elemental composition of the sample. Alizarin red images, scale bars represent 300 μm. XRF images, scale bars represent 900 μm. N = 3.

When added in the presence of growth medium (GM), levels of alkaline phosphatase (ALP) per cell were significantly decreased when compared with the BMP-2 gold-standard in both MO-EV and NMO-EV treated hBMSC cultures at days 4, 7 and 14 (Fig. 2a). An inverse pattern was found when EVs were administered in the presence of mineralisation medium (MM), with significantly elevated ALP levels observed in both MO-EV- and NMO-EV-treated hBMSC cultures at days 4 and 7 when compared with the MM control (Fig. 2b). In the presence of MM, cellular levels of ALP were not found to be significantly different in the EV treatment cultures compared to the BMP-2 gold-standard.

Alizarin red (AR) staining and X-ray fluorescence (XRF) elemental mapping revealed only limited calcium deposition when NMO-EVs and MO-EVs were added in the presence of growth medium (GM) (Fig. 2c and d). XRF maps when displayed in absolute mode, where the Ca and P intensities are normalised to the highest intensity elemental peak detected in the complete map, showed that calcium levels were enhanced if NMO-EVs were administered in the presence of MM when compared with GM. Visually, levels of calcium in these cultures resembled those observed the MM control and the BMP-2 gold-standard (Fig. 2d). The addition of MO-EVs in the presence of MM resulted in large areas of calcium deposition when visualised by AR staining and XRF under absolute mode (Fig. 2d). Observation of these cultures under relative mode (Fig. 2d row i), where the Ca and P intensities are normalised to the highest intensity of Ca and P in the map, highlighted areas of Ca and P co-localisation (yellow) were present only in MO-EV/MM-treated cultures. Calcium deposition in MO-EV/GM, NMO-EV/MM and GM, BMP-2 and MM cultures appeared to be diffuse and lacked the intensely fluorescent agglomerations observed in MO-EV/MM cultures (Fig. 2d row ii). Limited co-localisation of Ca and P was observed in these cultures suggesting that both elements were not associated with a mineral phase but rather as isolated components. Phosphorus was found to be ubiquitously expressed in all samples and was considered to largely identify the presence of phospholipids in the hBMSC cellular membranes (Fig. 2d row iv). However, visual differences in P were observed between hBMSCs exposed to GM and MM, with those exposed to MM displaying enhanced P intensity and linking these increases to the presence of mineral and a residual beta-glycerophosphate presence (Fig. 2d).

### sEVs derived from mineralising osteoblasts act as extracellular sites of mineral nucleation within the developing matrix

**Figure 3:**
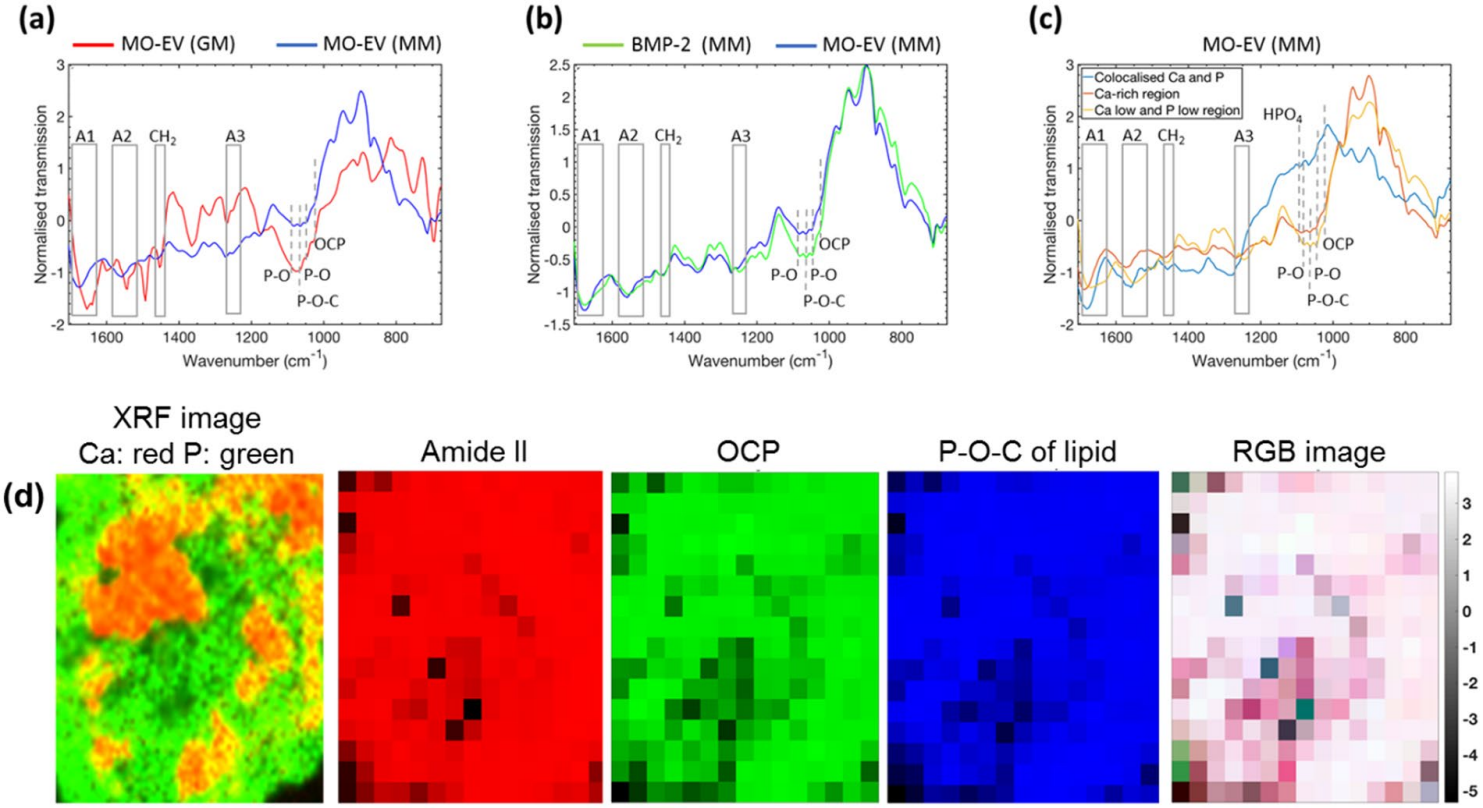
FTIR characterisation of organic and inorganic components of the hBMSC ECM. Pairwise comparison of FTIR spectra within the 675–1700 cm^−1^ spectral range using the standard normal variate (SNV) method for hBMSC cultures treated with (**a**) MO-EV/GM vs. MO-EV/MM, (**b**) BMP-2/MM vs. MO-EV/MM, and (**c**) regions within MO-EV/MM. Principal component analysis (PCA) was applied to define how mineral was associated with the ECM (**d**).

In order to define the organic and inorganic components of the ECM environment within the calcium-rich MO-EV/MM-treated hBMSC cultures we performed Fourier-transform infrared spectroscopy (FTIR) analysis within the 650–1700 cm^−1^ spectral range. The spectra were found to contain several vibrational bands representing components within a mineralising ECM, such as ECM proteins, lipids and P-O bonds of PO_4_^3−^ ions. Normalisation of the FTIR spectra using the Standard Normal Variate (SNV) method and auto-scaling of the spectra enabled comparison of relative peak heights across the samples while minimising the influence of varying sample thickness. The vibrational amide I (1650–1700 cm^−1^), amide II (1530–1575 cm^−1^) and amide III (centred at 1240 cm^−1^) indicative of ECM proteins were detected in both MO-EV samples (Fig. 3a)^28–31^. However, in hBMSC cultures dosed with MO-EV that had been administered in the presence of MM (MO-EV/MM), a significant enhancement in amide peaks was noted. This included broadening of amide I and II peaks, as well as an increased intensity in the amide III peak (Fig. 3a, blue line). Furthermore, the intensity of the amide peaks collectively relative to the P-O peaks was higher than that of MO-EV exposed to hBMSC cultures in the presence of growth media (GM) (Fig. 3a, red line), suggesting a higher abundance of matrix proteins. It should be noted that IR frequencies and their relative contribution within the amide band depends upon on collagen conformation and the level of hierarchical structure present – particularly in the case of collagen. We acknowledge some shift in amide bands that are likely to be indicative of conformational changes related to an increased presence of Ca^2+^ in MO-EV/MM-treated hBMSC cultures. Relative enhancement could also be observed in the stretching P-O bond of PO_4_^3−^ (1080, 1035–1045 cm^−1^). Further increases in the primary P-O-C absorption band (1060 cm^−1^) and a P = O bond of phospholipids (1250 cm^−1^) were observed, which was attributed to the presence of characterised sEV membrane components, such as phosphatidylserine (Fig. 3a)^30,31^. While amorphous calcium phosphate (ACP) and octacalcium phosphate (OCP) have vibrational modes within this region, OCP was ruled as being the dominant phase given that the most intense band for OCP is typically located around ~1025 cm^−1^ and this peak was found be to significantly weaker in these experiments compared to the 1060 cm^−1^ in question. ACP is usually characterised in FTIR spectra a by broad single peak spanning 900–1200 cm^−1^. While the spectra in our case exhibit significant peak broadening, fine peaks are clearly discernible within this region suggesting the presence of other compounds in addition to a possible ACP phase.

Pairwise comparison of FTIR spectra made between hBMSC cultures treated with MO-EV/MM and the BMP-2 gold standard revealed overlap between primary, secondary and tertiary amide peaks but highlighted a relative increase in presence of PO_4_ mineral phase and phospholipids (P-O-C, 1060 cm^−1^; P = O, 1250 cm^−1^) in MO-EV/MM samples (b). Notably, a small shoulder located at 1025 cm^−1^ indicative of OCP^30,32^ was present in both hBMSC cultures exposed to MO-EV/MM and BMP-2 (Fig. 3b).

At this stage we acknowledged the limitation of analysing a bulk FTIR spectrum of the MO-EV in MM culture given the clear evidence in the XRF maps that this culture exhibited a highly heterogeneous chemical composition, with large regions highly enriched in Ca with relatively little P and smaller areas of Ca and P colocalisation. The mean spectra of three sub-regions within the MO-EV/MM micro-XRF readout were compared. These comprised of regions of elemental Ca and P co-localisation, Ca-rich regions, and regions where there was little Ca or P (Fig. 3c). There was little difference in the inorganic composition of the regions low in Ca and P and those rich with Ca. The only features noted were enhancement of the amide I peak relative to P-O peaks and a broad weak shoulder forming between 1090–1150 cm^−1^ in the Ca-rich region. The regions of Ca and P colocalisation showed substantial decrease in the intensity of P-O, OCP and P-O-C peaks relative to the amide and CH_2_ peaks, although the OCP shoulder was still clearly visible. A new peak around 1092 cm^−1^ was identified along with some weak shoulder peaks between 1120–1132 cm^−1^, which are believed to correspond to HPO_4_^2−^. These ions are most likely to be within the OCP phase, but can also more generally relate to apatitic phases. The strong Ca signal in the micro-XRF maps yet weak FTIR peaks suggests that a sizable fraction of the Ca might be associated with organic matter at this early stage of mineralisation.

FTIR maps (relative mode) of the organic matter and OCP were generated in order to define how the mineral phase associated with the developing ECM in the MO-EV MM culture (Fig. 3d). The map of the protein matrix (red map) showed intense pixel intensity over the entire culture area. OCP (green map) did not map exclusively with the protein matrix and was found to correlate more strongly with the regions where the strong Ca was seen in the micro-XRF map. The signal from phospholipids (blue map) correlated very strongly with the presence of OCP, thus suggesting the developing mineral component was primarily associated with phospholipid and not directly associated with the collagenous matrix.

### The proteome of MO-EVs is enriched in binding proteins with roles in cell adhesion and extracellular matrix organisation

**Figure 4:**
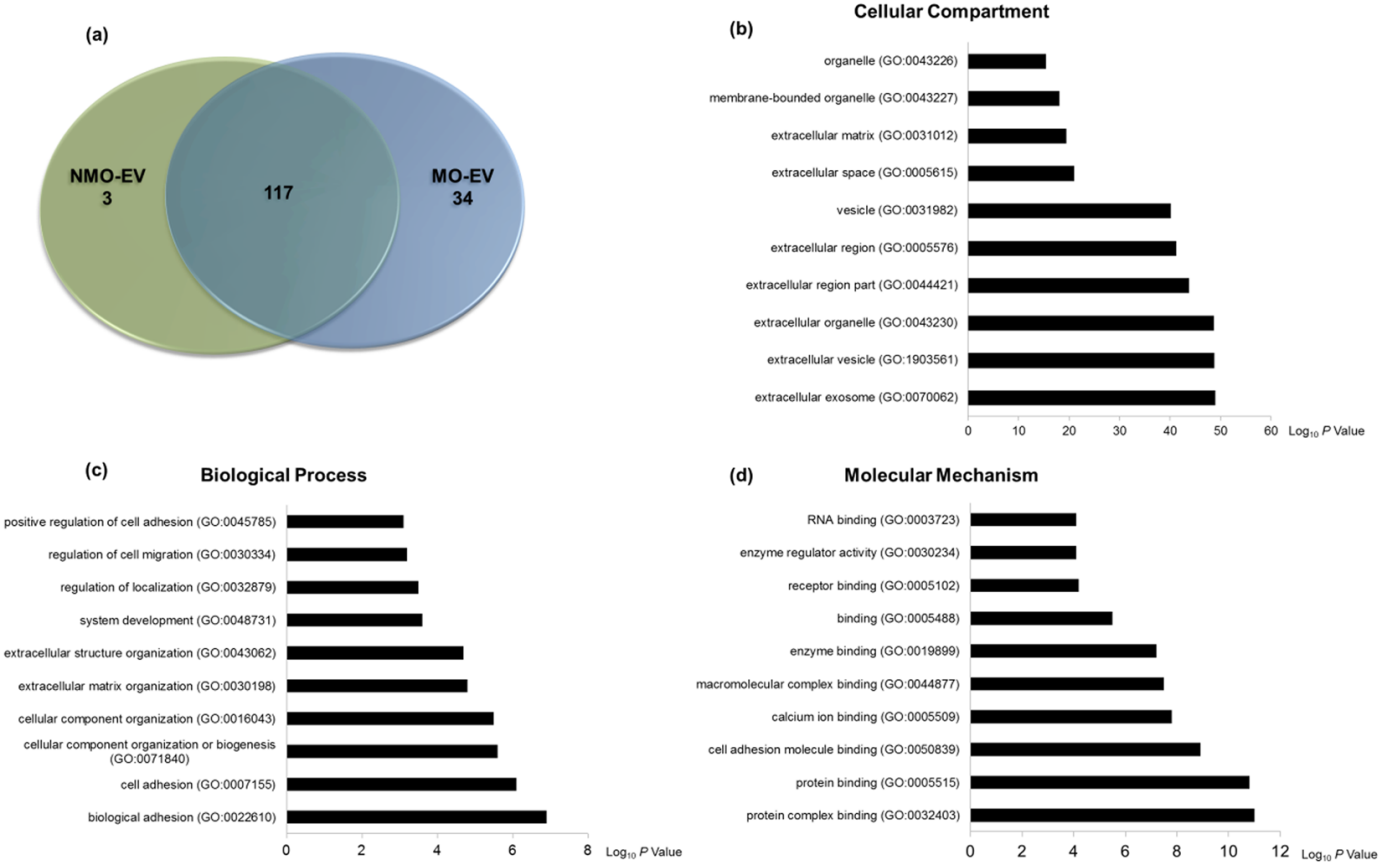
Gene ontology (GO) analysis of proteins found to be exclusive to or significantly upregulated in MO-EVs. (**a**) Venn diagram comparing the proteomes of MO-EVs with that of NMO-EVs identified a total of 117 shared proteins, 34 proteins exclusive to MO-EVs and 3 proteins that were exclusive to NMO-EVs. GO prediction of the (**b**) cellular compartment (**c**) biological function and (**d**) molecular mechanism of proteins significantly upregulated or exclusive to MO-EVs identified vesicular and extracellular matrix binding proteins with principal roles in cell adhesion and extracellular matrix organisation. The top ten most significant hits are presented for each GO designation. N = 3.

The proteomes of MO-EVs and NMO-EVs were compared for three independent sample preparations using a label-free MS-LC/LC approach. The use of stringent criteria only permitted the inclusion of proteins identified in at least two biological replicates, with >2 spectral counts (SC) in at least one repeat. Protein database searching resulted in the identification of a total of 154 proteins. Of these, 117 proteins were found to be shared between MO-EVs and NMO-EVs, 34 were specific to MO-EVs, and 3 were specific to NMO-EVs (Fig. 4a). A strong MS/MS peak intensity correlation was evident between the shared protein profiles of NMO-EVs and MO-EVs, with an average Pearson correlation coefficient of 0.899 (Supplementary information, S2).

Consistent with the derivation of the sEVs obtained in this study, both NMO-EVs and MO-EVs contained endosome-associated proteins, including Rab GTPases (Rab11a), common metabolic enzymes (Eno1, Pkm, GAPDH), Heat shock proteins (HSP90ab1, HSPa1a), membrane transport proteins (Anxa2, Anxa4, Anxa5, Anxa6), cytoskeletal components (Acta1, Actb, Tuba1b, Tuba4a, Tubb5, Cfl1), membrane adhesion proteins (Itgb3, Itga2b), signal transduction protein kinases (Marcks, Pgk1), common proteins related to apoptosis (histones), anti-apoptotic proteins (Lgals1), and the tetraspanin protein CD81. A comprehensive list of common EV marker proteins and their relative abundance in NMO-EV and MO-EV samples can be found in the Supplementary information (S2). Specifically, this study highlighted possible differences in commonly used, yet not fully standardised EV surface membrane marker proteins between NMO-EVs and MO-EVs. Although further analysis, which lies outside the scope of the present study, will be required to confirm these differences, they may represent an effective way to select for vesicles that have the capacity to induce matrix mineralisation. These proteins comprised of members of the tetraspanin protein family (CD9), programmed cell death 6 interacting protein (PDCD6ip) and heat shock protein 90 alpha family class A member 1 (HSP90aa1).

To provide an overview of the principal processes, mechanisms and cellular location of proteins exclusive to or significantly upregulated in MO-EVs Gene Ontology (GO) analysis was performed. Cellular compartment prediction confirmed that these proteins were confined to EVs and extracellular environment (Fig. 4b). Biological process analysis indicated that these proteins primarily functioned in cell adhesion and organisation of the extracellular matrix (Fig. 4c). Molecular mechanisms of these proteins, included protein- and calcium ion-binding (both calcium-dependent and calcium-independent), as well as roles in the binding of other membrane-associated molecules, such as lipids and phospholipids (Fig. 4d).

### Calcium-binding proteins, osteoblast-bridging collagens and regulators of ECM mineralisation are implicated in the potent osteoinductive effects of MO-EVs

**Figure 5:**
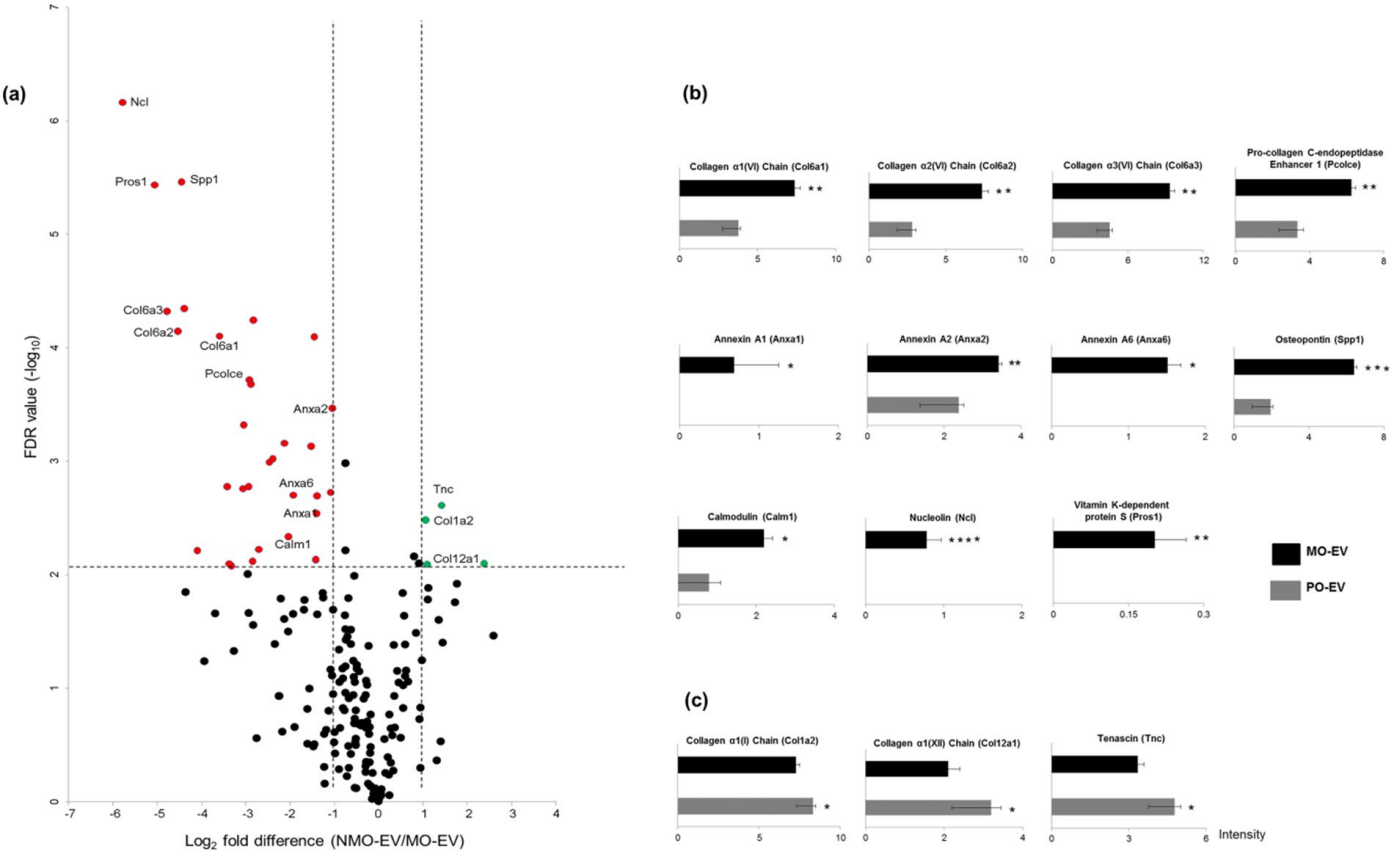
Analysis of differentially expressed proteins identified using LC-MS/MS between MO-EVs and NMO-EVs. (**a**) Volcano plot displaying Log_2_ values for protein fold-change against Log_10_ FDR. Proteins with a Log_2_ fold difference below 1 and a statistical value of >0.05 (horizontal dashed line) were not considered to be statistically significant. Proteins found to be significantly upregulated in MO-EVs are displayed in red. Proteins that were significantly upregulated in NMO-EVs are displayed in green. Graphical representations of LC-MS/MS signal intensity plots for proteins found to be differentially expressed within (**b**) MO-EVs and (**c**) NMO-EVs. *^,^ **^,^ *** and **** signify *P* values of <0.05, <0.01, <0.001 and <0.0001, respectively. N = 3.

Lastly, we sought to further define proteins that may be responsible for the potent osteoinductive effects of MO-EVs. Proteins identified by LC-MS/MS were compared by correlating differences in Log_2_ values of fold-protein change between MO-EVs with NMO-EVs and plotting these against the false discovery rate (FDR). Of the total number of proteins found to be significantly upregulated in MO-EVs, 27 of these exhibited a fold-change of >2. Application of these stringent selection criteria was applied to distinguish only the most significant proteins likely to be associated with MO-EV enhanced hBMSC mineralisation (Fig. 5a). The 27 proteins identified as being significantly up-regulated in MO-EVs, included cytosolic proteins involved in signal transduction (Sdcbp, P < 0.05), osteogenesis (Spp1, P < 0.001), collagen modification (Pcolce, P < 0.01), calcium binding (Anxa-1, P < 0.05; Anxa-2, P < 0.01 and Anxa-6, P < 0.05), intracellular ATP generation (Nme2, P < 0.05), bridging collagens involved in osteoblast interactions (collagen type-VI α1, 2 and 3, P < 0.01), the calcium-binding messenger protein (Calm1, P < 0.05), a major nucleolar protein (Ncl, P < 0.0001), and the vitamin K-dependent protein S (Pros1, P < 0.001). A comparison of the relative MS/MS peak intensities of proteins considered to be most related to MO-EV mineralisation are presented in Fig. 5b. A comprehensive list delineating all proteins significantly up-regulated in the MO-EV fraction can be found in the supplementary information. In contrast, only 4 proteins were found to be significantly (P < 0.05) upregulated in NMO-EVs. These consisted of the extracellular matrix glycoprotein tenascin (Tnc), histone H1.3 (Hist1h1d), collagen type-I alpha2 (Col1a2) and collagen type-XII alpha1 (Col12a1). A full list of proteins identified as specific to- or significantly upregulated in MO-EVs is presented in the Supplementary information (S3). Of note is the fact that phosphatase enzymes, alkaline phosphatase (ALP) and ectophosphodiesterase nucleotide pyrophosphatase (enpp) were not identified on either NMO-EVs or MO-EVs.

## Discussion

The majority of investigations into the osteoinductive effects of osteoblast-derived vesicles have been confined to those bound within the ECM (MVs). Relatively few studies have examined the roles of small extracellular vesicles released into the culture medium (sEVs). From a therapeutic perspective EVs have considerable benefits over MVs as they can be simply isolated without the need for time consuming ECM digestion that could potentially have a negative effect on the activity of any bioactive materials within them. In the present study we sought to determine whether osteoblast-derived sEVs could be utilised as an acellular yet biologically inductive method for inducing human stem cell differentiation and define the underlying biochemical mechanisms involved.

The present study provides comprehensive evidence to show that the culture environment in which osteoblast-derived sEVs are generated is of critical importance if they are to be successfully applied to induce hBMSC differentiation towards a mineralising phenotype. Significant differences in the capacity of sEVs to induce hBMSC mineralisation was observed between NMO-EVs and MO-EVs, with only the administration of MO-EVs in the presence of MM found to greatly enhance the deposition of calcium in the surrounding ECM (Fig. 2). The fact that MO-EVs only enhanced matrix mineralisation in the presence of MM led to the hypothesis that sEVs continued to accumulate calcium and phosphorus ions required for mineralisation within the developing ECM. The question of whether osteoblast-derived vesicles are preloaded with calcium and phosphorus at the time of their release or whether these vesicles accumulate ions extracellularly has long been of interest^33^. There is a collective body of evidence to demonstrate that both mechanisms occur *in vivo*, with the presence of both electron lucent and electron dense sEVs observed at progressing stages of matrix mineralisation. Distinctions in the genesis of vesicle subtypes lend further support to these findings. For instance, small endocytic vesicles termed exosomes have been shown to accumulate Ca^2+^ and P intracellularly through association with organelles, such as the mitochondria, while the extracellular accumulation of Ca^2+^ and PO_4_^3−^ ions has been posited to be a feature of micro-vesicles that bud directly from the cellular plasma membrane and subsequently associate with the collagenous ECM^34^. Here, TEM evidence demonstrates that sEVs derived from the medium of mineralising osteoblast cultures facilitate the transport of electron-dense material (Fig. 1e). However, it is hypothesised that a large subsection of these unbound vesicles also continue to accumulate ions extracellularly and that this is critical for the sEV-induced mineralisation of hBMSC cultures. This data acts to highlight heterogeneity in the sEV population, which was fractioned based on differential centrifugation and will certainly comprise of exosomes as well as larger microvesicles. Given that the present study has provided evidence to show that a heterogeneous population of MO-EVs is efficacious for inducing hBMSC mineralisation, an important next step will be to separate exosomes from small micro-vesicles to determine the independent osteoinductive effects of each sub-population on stem cells. Furthermore, LC-MS/MS data presented in the present study suggested that, unlike matrix-bound vesicles, both NMO-EVs nor MO-EVs derived from the culture medium lacked phosphatase enzymes required to process exogenous beta-glycerophosphate added to the culture environment (Supplementary Figure, S3). This led us to conclude that enhanced cellular ALP expression identified in our study was essential for facilitating the generation of inorganic phosphates that could associate with MO-EVs within the extracellular environment, co-localising with calcium to drive mineral formation.

Although evidence implicating EVs as an initial site of early mineralisation within developing bone has long been postulated, the mechanisms by which EVs initiate matrix mineralisation are not entirely clear^12,13^. There is evidence identifying vesicle association with the collagenous ECM and the subsequent deposition of an amorphous calcium phosphate (ACP) as a potential mechanism for advancement of the mineralisation front within the growth zones of developing bone^35^. Although, the biochemical processes governing the formation of biological apatite are not fully understood, more soluble calcium phosphate phases have been implicated as precursors in its formation. Evidence has existed since 1957 to suggest that these are subsequently transformed into a more stable and less soluble apatitic phase in a thermodynamically favoured process^36^. However, much debate still surrounds this hypothesis and, to date, evidence has only been provided by a small number of *in vivo* studies, such as the evolving zebrafish bone model^35^. The present study provides evidence of a transitioning mineral phase, identified using FTIR as octacalcium phosphate (OCP), in hBMSC exposed to MO-EVs after just 14 days culture (Fig. 3). Metastable OCP has long been regarded as near-terminal transitional phase in the formation of biological apatite that occurs only under more acidic conditions^37^. Using micro-XRF and FTIR mapping combined with statistical processing of the spectra (Fig. 3d), we were able to deduce that rather than being principally associated with the collagenous ECM, OCP was localised to the P-O-C component of phospholipid, known to be enriched in the EV membrane^30,31,38^. The continued association of this transitioning mineral component with the EV membrane within the ECM would indicate that early mineral nucleation largely occurs in association with the phospholipid membrane of the maturing EVs. This hypothesis is further supported by previous studies that have shown EVs are typically found to be of a more acidic pH, facilitating fusion with the membrane of recipient cells^39,40^ and limiting the dissolution of metastable OCP. However, further experimental research is required to elucidate the process of mineral nucleation and crystallisation on the phospholipid-rich membrane of EVs. Currently, evidence required to convincingly demonstrate the transition of mineral to a stable apatite from an amorphous precursor is lacking due to the transience of each phase during *in vivo* osteogenesis that is further confounded by difficulties in visualising these mineral phases^41^.

In order to further support the hypothesis that mineral nucleation is initially confined to the phospholipid-rich inner membrane of sEVs, the entire protein component of MO-EVs and NMO-EVs were profile to identify proteins required for the chelation of mineral ions and the advancement of mineral formation (Fig. 5). Annexins represent a well characterised group of Ca^2+^ chelators that interact with phospholipids, including phosphatidylserine (PS) within the inner EV membrane to from PS-Ca^2+^-P complexes, which act as a centre for the crystallisation and nucleation of mineral on an osteoid template^42^. It has previously been shown that the presence of OCP was largely associated with the EV phospholipid membrane, which is thought to act in conjunction with calcium chelating proteins and orthophosphate as an intraluminal site for mineral nulecation^43^. In the present study it is shown that annexins A1, A2 and A6 are significantly upregulated in MO-EVs when compared with NMO-EVs, contributing to an inner nucleational core that is, at least in part, likely to be responsible for the delivery and propagation of mineral within the surrounding matrix of hBMSC cultures^44,45^. Previous studies have described the presence and enrichment of annexins within matrix-bound vesicles (MVs) and their importance in events such as pathological mineralisation is well demonstrated^46^. However, the presence of these Ca^2+^ binding proteins in osteoblast-derived EVs has been described by only a limited number of groups^26^. Within osteoblast cultures, an increase in the expression of annexin I has been associated with enhanced ALP activity and PTH-induced cAMP stimulation^47^. Subsequent analysis of the role of annexins in osteoblast-mediated mineralisation identified that these increases in alkaline phosphatase activity were localised to lipid rafts that subsequently become internalised within endosomes and exported in small EVs (<200 nm) called exosomes^48,49^. However, the significant roles that these calcium-binding proteins may play during bone formation, on the whole, been largely overlooked^47^. Given our findings it is possible that significant up-regulation of annexin proteins observed in MO-EVs is likely to have an important role in binding of extracellular calcium ions, acting as a site for mineral nucleation and contributing to the significantly increased mineralisation status of hBMSC cultures in the presence of MM.

In addition to an increase in the presence of Ca^2+^ chelating annexin proteins, evidence is presented to indicate that the type of collagen associated with EVs is temporally dependent on the mineralisation status of the osteoblast (Fig. 5). Given their size, these larger ECM components are thought to be anchored to EVs via various binding proteins rather than being localised within the EV lumen^50^. Both vesicles-derived from mineralising and non-mineralising osteoblasts were found to contain collagen-binding proteins, such as protein disulphide isomerase, which may serve to anchor collagens to the EV and provide a mechanism of transit from the endoplasmic reticulum to the ECM^51^. Intriguingly, MO-EVs were found to be significantly enriched with collagen type-VI but were less associated with collagens type-Ia and -XII when compared with NMO-EVs. The contribution of collagen type-I to mineralisation is well described and it represents the major ECM component of bone. However, osteogenesis is a complex process that requires the contribution of other structurally distinct, and frequently overlooked, collagens. Collagen type-VI (non-fibrillar) and -XII (fibrillar) have coordinated roles in the formation of collagen bridges involved in osteoblast cell-cell communication, with the deletion of these genes leading to a reduction in bone mass and increased fragility^52^. Collagen-VI is a primary component of the periosteum ECM that is thought to function in early osteoblast differentiation and formation of the primary osteon^53,54^. Receptors for collagen-VI are found on the surface of chondrocytes and have been shown to transduce signals to the mitochondria and having important roles in the release of mitochondrial Ca^2+^, which is subsequently packaged into endosomal vesicles and delivered into the extracellular environment^55^. Collagen-XII contains several domains, specifically one collagenous domain that interacts with collagen-I and another large N-terminal globular domain that has been shown to interact with molecules, such as tenascin, which regulate cellular interaction with the surrounding pericellular matrix, influencing osteoblast adhesion and differentiation, and which was found here to be significantly upregulated in NMO-EVs^56^. To our knowledge, this study presents the first indication that the deposition of these bridging collagens is, at least in part, coordinated by the extracellular delivery of osteoblast-derived EVs and that the transfer of these collagens is likely temporally distinct.

Other proteins that were found to be significantly up-regulated in MO-EVs also warrant some brief discussion, since their contribution may have important implications for early mineralisation that demand further investigation. The majority of these proteins function in the binding of calcium ions and as intracellular messengers but have under characterised roles during early osteogenesis. These proteins included nucleolin, calmodulin and protein-S. Nulceolin is a nuclear phosphoprotein that has previously been shown to be translocated in small matrix vesicles to the cell surface^57^. Although the relationship between nucleolin and osteogenesis remains unknown, there is evidence to suggest this calcium-binding protein is linked to cartilage matrix degradation through MMP-9 and the relaying of signals to the ECM and nucleus^58^. A second calcium-binding protein found to be up-regulated in MO-EVs was calmodulin, which is activated through the binding of calcium ions and functions in the Ca^2+^ signal transduction pathway that enhances osteoblast differentiation and bone development^59^. Finally, a protein that was found to be significantly up-regulated in MO-EVs yet largely uncharacterised in relation to osteogenesis is the vitamin-K-dependent protein, protein-S. Unlike nucleolin and calmodulin, protein-S does not function in the binding of calcium ions. Members of the vitamin-K-dependent protein family have well defined roles in osteogenesis as well as in pathological vascular calcification. Currently, little information exists regarding the role of protein-S during osteogenesis. However, it has previously been hypothesised that this protein may be as important as other well characterised vitamin K-dependent proteins, such as osteocalcin and matrix gamma-carboxyglutamic acid (Gla) protein, in bone turnover^60^. It is recommended that further examination of this protein is required in order to better define its role during early osteogenesis.

In conclusion, data is provided to show that sEVs derived from mineralising osteoblasts have considerable utility as an acellular approach to mineralised tissue engineering. Interestingly, the contents of these vesicles appear to be dependent on the culture environment in which they are generated, with sEVs derived from non-mineralising osteoblasts demonstrating limited efficacy *in vitro*. Comparative proteomic analysis of MO-EVs and NMO-EVs highlighted significant up-regulation in the presence of calcium chelating proteins, namely annexins, that have been shown to have important yet under-characterised roles in the EV-localised mineral nucleation, as well as bridging collagens previously shown to be important in the formation of the early mineralising ECM. Lastly, since the mineral identified was defined as OCP and found to be primarily associated with phospholipid, it is proposed that this likely represented a transitioning mineral phase that continued to remain in contact with EVs even within the developing ECM. Perhaps one of the most striking findings, and a further reason to hypothesise that EVs acted as an early site of mineral nucleation, was that MO-EVs were only capable of significantly enhancing mineralisation when added in the presence of MM. This suggests that exogenous ions within the medium became associated with the MO-EVs extracellularly - likely due to the enhanced presence of annexins - and were required for the formation of OCP in these cultures.

## Materials and Methods

### Cell Culture and Reagents

Human bone marrow-derived mesenchymal stem cells (hBMSCs) were purchased from Lonza (Lonza, UK http://www.lonza.com). hBMSCs were fully profiled for the expression of CD29, CD44, CD105 and CD166 and lack of CD14, CD34, CD45. MC3T3 murine osteoblasts were purchased from America Type Culture Collection (ATCC, http://www.atcc.org). Cell culture medium comprised of minimal essential medium (α-MEM; Sigma-Aldrich, UK) supplemented with 10% foetal bovine serum (FBS), L-glutamine (Sigma-Aldrich, UK) and 1% penicillin/streptomycin (Sigma-Aldrich, UK). hBMSCs were used between passage 4–5. Mineralisation medium was formulated through the addition of 10 mM β-glycerophosphate (Sigma-Aldrich, UK) and 50 μg/mL L-ascorbic acid (Sigma-Aldrich, UK). Culture medium used for EV isolation was depleted of any contaminating EVs by ultra-centrifuging the FBS at 120,000 g for 70 minutes prior to use.

### Exosome Isolation and Characterisation

MC3T3 osteoblasts were cultured at scale in T175 culture flasks (Nunc, UK) and the medium isolated every two days. Osteoblasts were grown in either standard culture medium or osteogenic medium for a total period of 14 days. Osteoblast-derived EVs were isolated from collected medium by differential centrifugation: 2000g for 20 minutes to remove cell debris and apoptotic bodies, 10,000 g for 30 minutes to remove micro-vesicles, 120,000 g for 70 minutes to pellet EVs. Following the final ultracentrifugation step, the supernatant was removed, the pellet washed in sterile phosphate buffered saline (PBS) and further centrifuged at 120,000 g. All ultracentrifugation steps were performed using a Sorvall WX Ultra Series Ultracentrifuge (Thermo Scientific, UK) with a Fiberlite, F50L-8 × 39 fixed-angle rotor (Piramoon Technologies Inc., USA).

The resulting pellet was re-suspended in 200 μL of PBS and the total protein concentration determined using the Pierce BCA protein assay kit (Thermofisher Scientific, UK, http://www.thermofisher.com). Particle size distribution was analysed using Dynamic Light Scattering (DLS; Malvern Instruments, UK) and quantitated using resistive pulse sensing (IZON qNano) and nanoparticle tracking analysis (Nanosight LM10; Malvern Instruments, UK).

### Atomic Force Microscopy

5 μL of EV suspension was adsorbed onto freshly cleaved mica sheets and left to dry at 4 °C overnight. Samples were rinsed with deionised water and left to dry naturally in a flow hood. The presence of exosomes was determined using a NanoWizard II atomic force microscope (JPK Instruments, Germany) under tapping mode using FESP cantilevers (Bruker, Germany). Images were acquired using a scan rate of 0.25 Hz. Image processing was performed using JPK data processing software.

### Transmission Electron Microscopy

A suspension of EVs was made in sterile PBS. A single drop of the suspension (~20 μL) was deposited onto a carbon film grid (Agar Scientific, UK) and left to air dry for a period of 1 minute. To remove excess PBS the grid was gently blotted using tissue paper through capillary action. Negative staining was achieved by placing a single drop (~20 μL) of uranyl acetate solution onto the dry grid. Samples were imaged using a JEM 3200FX transmission electron microscope (Joel, USA) using a voltage of 80 kV.

### Stem cell culture in the presence of EVs

hBMSCs were seeded in 6 well culture plates (Nunc, UK) at a density of 21 × 10^3^ cells per cm^2^ and incubated at 37 °C, 5% CO_2_ to allow cell attachment. After 24hrs the medium was replaced with EV-free medium in the presence and absence of mineralisation suppliments, 10 mM β-glycerophosphate (Sigma-Aldrich, UK) and 50 μg/mL L-ascorbic acid (Sigma-Aldrich, UK). EVs were added to each well at a concentration of 10 μg/mL. Medium changes and EV replenishment was performed every 48 hrs. As a positive control, MSCs were exposed to mineralisation medium in the absence of EVs. As a gold-standard comparison, MSCs were cultured in the presence of BMP-2 and MM, since BMP-2 is a commercially available and clinically applied growth factor shown to induce mineralisation *in vitro* ^61^ and in instances of non-union and spinal fusion^62^.

### Alkaline Phosphatase Assay

Cellular alkaline phosphatase (ALP) levels were quantified using the SensoLyte® pNPP Alkaline Phosphatase Assay Kit (AnaSpec, USA) according to the manufacturer’s instructions. Briefly, cell monolayers were washed twice using 1x assay buffer provided. Cells were detached from the surface of the culture plate in the presence of 200 μL permeabilisation buffer using a cell scraper. The resulting cell suspension was collected in a microcentrifuge tube, incubated at 4 °C for 10 minutes under agitation, and then centrifuged at 2500 g for 10 minutes. Supernatant was transferred to a 96 well plate where it was combined with an equal volume of pNPP substrate. Absorbance was measured at 405 nm using a GloMax Multi Plus spectrometer (Promega, UK).

### Alizarin Red Staining and Quantification

Calcium-rich deposits were visualised using 40 mM alizarin red (Sigma-Aldrich, UK) stain, which was dissolved in acetic acid and adjusted to pH 4.2 using 5 M ammonium hydroxide.

### Micro-XRF mapping of cell cultures

Samples for analysis were prepared by transferring mineralised cell monolayers onto an aluminium foil covered glass slide and leaving to dry overnight at room temperature. Maps of calcium and phosphorous across dried cell culture media spot samples were acquired using a Tornado M4 micro-XRF system (Bruker Nano, Germany) fitted with a Rhodium micro focus X-Ray tube and a polycapillary lens, with the X-ray tube set to 50 kV voltage and 300 μA current. The chamber pressure was lowered to 20 mbar in order to maximise sensitivity to the phosphorus signal. The system was programmed to acquire a map of the whole slide containing all of the media spot samples by rastering the microfocus beam over the slide with a step size of 30 μm and a time per pixel of 30 ms. An XRF spectrum was collected at each pixel and elemental maps generated progressively in real time by gating around the phosphorous K_α1_ (2.0137 keV) and calcium K_α1_ (3.692 keV) X-Ray fluorescence peaks in the spectra, creating an image where pixel intensity represented X-Ray detector pulses per eV at each measurement point on the sample. Maximum pixel intensity for each element was normalised to the peak pulses/eV value for that element across the whole sample.

### Infrared mapping of EV-treated hBMSCs

Cell monolayers were carefully transferred from culture dishes and spotted on to an aluminium coated glass slide. Infrared spectroscopy (IR) maps of each spot were acquired using a Nicolet iN10 MX Infrared Imaging Microscope, coupled to a liquid nitrogen cooled MCT-A Detector with a CdTe window detector, and operated in reflectance mode. The spectral range acquired was 4000 to 675 cm^−1^ with a spectral resolution of 4 cm^−1^, and 64 acquisitions acquired for each spectrum. The aperture size was 150 × 150 μm with x and y step sizes of 150 μm. A gold standard was used in spectral background calibration.

### Pre-processing of hyperspectral datacubes

Each data file contained a hyperspectral datacube consisting of (x,y) pixel locations across the sample and a third dimension containing a vector of measured absorbance values at each wavenumber. Each vector therefore represented the averaged IR spectrum for pixel x,y as collected by the instrument. The hyperspectral datacube was imported into MATLAB (MATLAB R2016b, Mathworks, Natick, Massachusetts, USA) for pre-processing and analysis using in-house developed code. Each spectrum in the data cube was normalised using the Standard Normal Variate (SNV) method, which aids comparison between spectra. SNV normalisation processes the spectra such that they are collectively mean centred and divided by their own standard deviation as follows:

1 $${\boldsymbol{SNV}}\,({\boldsymbol{Spectrum}})=\frac{{\boldsymbol{Spectrum}}-\bar{{\boldsymbol{x}}}}{{\boldsymbol{s}}}$$

$\bar{{\rm{x}}}$ = spectrum mean, s = spectrum sample standard deviation.

The resulting spectra had a mean value of zero and a standard deviation of one. Autoscaling was then performed between the equivalently measured wavenumbers of the normalised spectra as follows:

2 $${\boldsymbol{Autoscale}}\,({\boldsymbol{Wavenumber}})=\frac{{\boldsymbol{Wavenumber}}-\bar{{\boldsymbol{x}}}}{{\boldsymbol{s}}}$$

$\bar{{\rm{x}}}$ = mean of all measured values at wavenumber (between spectra), s = sample standard deviation of all measured values at wavenumber (between spectra).

After autoscaling, a final pre-processed spectrum represented measured IR absorbance/transmittance values below the mean of all spectra as negative values, and IR absorbance/transmittance values above the mean as positive values.

### Peak deconvolution

Overlapping IR peaks were deconvoluted into constituent peak shapes and peak locations estimated through processing in MATLAB. Briefly, the spectra were clipped to include only the spectral range where the merged peaks were located and background corrected using a linear background approximation. An initial guess on the number of Gaussian and/or Lorentzian distributions and their spectral centres to fit to the data was made through visual inspection of the spectra. An unconstrained non-linear optimisation algorithm was employed to decompose the spectra and optimise the shape and location of the peaks. The objective was to minimise the fit error% and maximise R^2^. The deconvolution process was run multiple times and sometimes the initial guess of the number of peaks to fit was changed against visual intuition as a way of searching for potentially more optimal fits.

### Mass Spectrometry

Exosome proteins were digested by FASP with LysC and trypsin as described previously^63^.

The peptides generated were then separated by nano-flow reversed-phase liquid chromatography coupled to Q Exactive Hybrid Quadrupole-Orbitrap mass spectrometer (Thermo Scientific, UK). Peptides were loaded on a C18 PepMap100 pre-column (300 μm I.D. × 5mm, 3 μm C18 beads; Thermo Scientific) and separated on a 50 cm reversed-phase C18 column (75 μm I. D., 2 μm C18 beads). Separation was conducted with a linear gradient of 7–30% B 120 min at a flow rate of 200 nL/min (A: 0.1% formic acid, B: 0.1% formic acid in acetonitrile). All data was acquired in a data-dependent mode, automatically switching from MS to collision-induced dissociation MS/MS on the top 10 most abundant ions with a precursor scan range of 350–1650m/z. MS spectra were acquired at a resolution of 70,000 and MS/MS scans at 17,000. Dynamic exclusion was enabled with an exclusion duration of 40 s. The raw data files generated were processed using the MaxQuant (version 1.5.0.35) software, integrated with the Andromeda search engine as described previously^64,65^.

The MS/MS spectra were searched against the mouse proteome (UniProt 2013/04/03), precursor mass tolerance was set to 20 ppm, variable modifications defined as acetylation (protein N-terminus) and oxidation (M), carbamidomethylation (C) was defined as fixed modification. Enzyme specificity was set to trypsin with a maximum of 2 missed cleavages. Protein and peptide spectral matches (PSM) false discovery rate (FDR) was set at 0.01 and match between runs was applied. Finally, only proteins identified in 2 biological replicates with a minimum of 2 spectral counts (SC) in one biological replicate were included for analysis. For proteins to be considered specific to either the MO-EV or NO-EV fraction they must have exhibited SC ≥ 5.

Differential protein abundance analysis was performed with Perseus (version 1.5.5.3). In brief, Log_2_-transformed protein intensity distributions were median-normalised and a t-test was used to assess the statistical significance of protein abundance fold-changes. P-values were adjusted for multiple hypothesis testing with the Benjamini-Hochberg correction^66^.

### Bioinformatic Analysis

The protein annotation through evolutionary relationship (PANTHER, http://www.pantherdb.org/) classification system (version 9.0) was used for gene ontology (GO) annotation of biological pathways, molecular mechanisms and cellular compartments of proteins found to be significantly upregulated or specific to MO-EVs.

## Electronic supplementary material

Supplementary Figures 1 and 2

Supplementary Figures 3 and 4
