# Supplementary material for: Annexin-enriched osteoblast-derived vesicles act as an extracellular site of mineral nucleation within developing stem cell cultures
Source: Sci Rep. 2017 Oct 3;7:12639. doi: 10.1038/s41598-017-13027-6 (PMC5626761; doi:10.1038/s41598-017-13027-6)
Supplement: Supplementary file 1 — Supplementary Figures 1 and 2 [file 41598_2017_13027_MOESM1_ESM.doc]

**Annexin-enriched osteoblast-derived vesicles act as an extracellular site of mineral nucleation within developing stem cell cultures**

*OG Davies^1,2^, SC Cox^2^, RL Williams^2^, D Tsaroucha^2^, R Dorrepaal^3^, MP Lewis^1^, LM Grover^2^

OG Davies, [O.G.Davies@lboro.ac.uk](mailto:O.G.Davies@lboro.ac.uk), [O.Davies@bham.ac.uk](mailto:O.Davies@bham.ac.uk), +44 (0)7885585953

^1^ School of Sport, Exercise and Health Sciences, Loughborough University, Epinal Way, Loughborough, LE11 3TU

^2^ School of Chemical Engineering, University of Birmingham, Edgbaston, Birmingham, B15

2TT

^3^ UCD School of Biosystems and Food Engineering, University College Dublin, Belfield, Dublin 4

**Supplementary Information**

**Supplementary 1 (S1).** Nanoparticle tracking analysis

Nanosight (LM10; Malvern Instruments, UK) image demonstrating the presence of EVs isolated from mineralising osteoblasts.


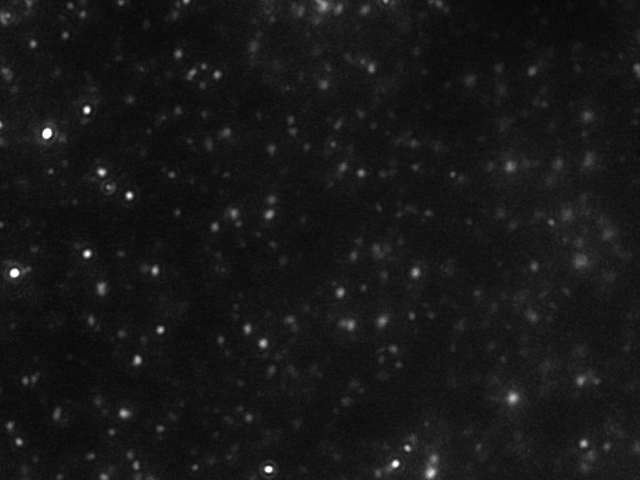


**Supplementay 2 (S2).** Pearson correlation

Plot depicting the correlation in LC-MS/MS peak intensity for three independent sample preparations of NMO-EV and MO-EV samples. An average correlation coefficient value of 0.899 was obtained from the collective datasets.


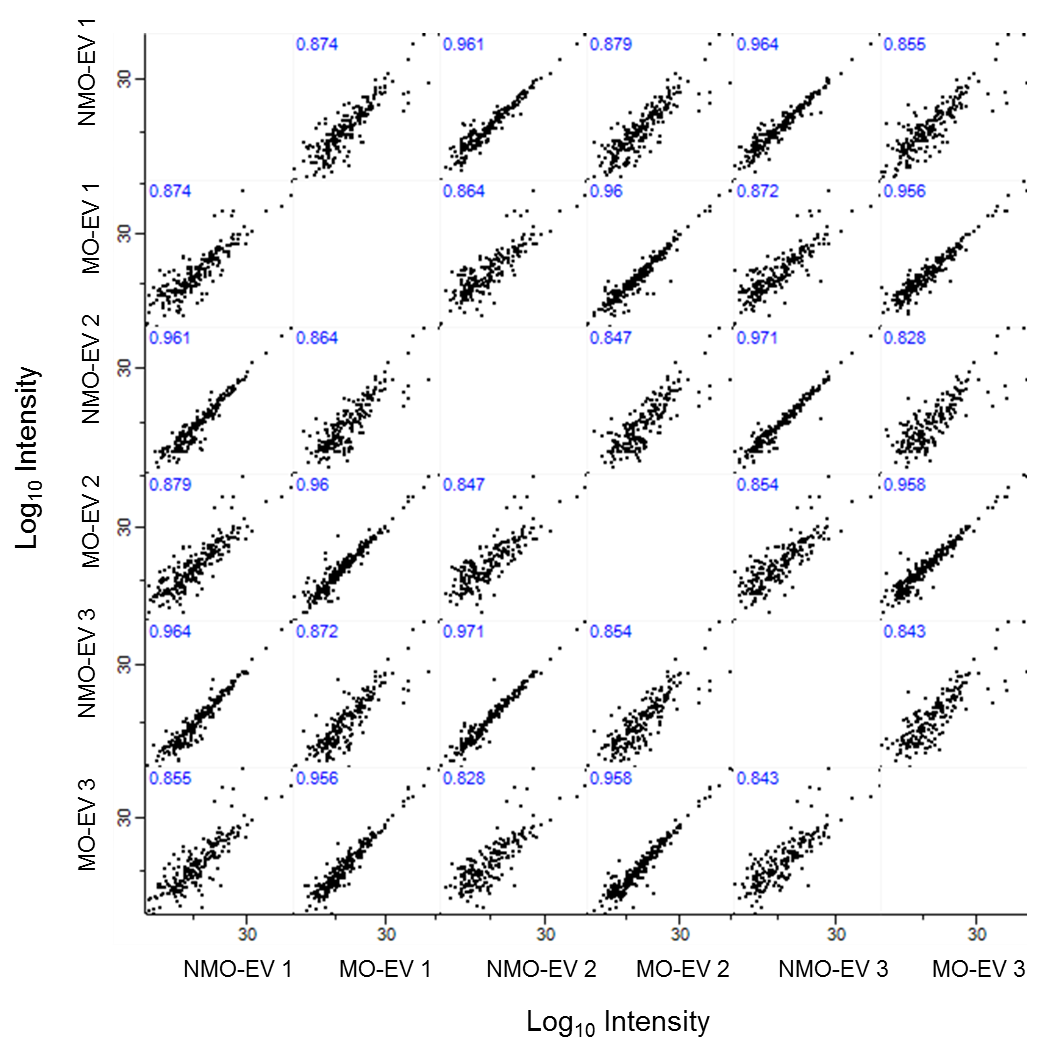


**Supporting Information Available**

S1. Nanosight (LM10; Malvern Instruments, UK) video image demonstrating the presence of EVs isolated from mineralising osteoblasts.

S2. Plot depicting the correlation in LC-MS/MS peak intensity for three independent sample preparations of NMO-EV and MO-EV samples.

S3. Complete list of proteins identified by LC-MS/MS.
